# Facilitating Surveillance of Pulmonary Invasive Mold Diseases in Patients with Haematological Malignancies by Screening Computed Tomography Reports Using Natural Language Processing

**DOI:** 10.1371/journal.pone.0107797

**Published:** 2014-09-24

**Authors:** Michelle R. Ananda-Rajah, David Martinez, Monica A. Slavin, Lawrence Cavedon, Michael Dooley, Allen Cheng, Karin A. Thursky

**Affiliations:** 1 Infectious Diseases Unit, Alfred Health, Melbourne, Victoria, Australia; 2 Computing and Information Systems, University of Melbourne, Melbourne, Victoria, Australia; 3 Victorian Infectious Diseases Service, Peter Doherty Centre, Melbourne, Victoria, Australia; 4 Infectious diseases department, Peter MacCallum Cancer Institute, Melbourne, Victoria, Australia; 5 School of Computer Science and IT, RMIT University, Melbourne, Victoria, Australia; 6 Pharmacy Department, Alfred Health, Melbourne, Victoria, Australia; 7 Faculty of Pharmacy & Pharmaceutical Science, Monash University, Melbourne, Victoria, Australia; 8 Department of Epidemiology and Preventative Medicine, Monash University, Melbourne, Victoria, Australia; Ospedale Pediatrico Bambino Gesu', Italy

## Abstract

**Purpose:**

Prospective surveillance of invasive mold diseases (IMDs) in haematology patients should be standard of care but is hampered by the absence of a reliable laboratory prompt and the difficulty of manual surveillance. We used a high throughput technology, natural language processing (NLP), to develop a classifier based on machine learning techniques to screen computed tomography (CT) reports supportive for IMDs.

**Patients and Methods:**

We conducted a retrospective case-control study of CT reports from the clinical encounter and up to 12-weeks after, from a random subset of 79 of 270 case patients with 33 probable/proven IMDs by international definitions, and 68 of 257 uninfected-control patients identified from 3 tertiary haematology centres. The classifier was trained and tested on a reference standard of 449 physician annotated reports including a development subset (n = 366), from a total of 1880 reports, using 10-fold cross validation, comparing binary and probabilistic predictions to the reference standard to generate sensitivity, specificity and area under the receiver-operating-curve (ROC).

**Results:**

For the development subset, sensitivity/specificity was 91% (95%CI 86% to 94%)/79% (95%CI 71% to 84%) and ROC area was 0.92 (95%CI 89% to 94%). Of 25 (5.6%) missed notifications, only 4 (0.9%) reports were regarded as clinically significant.

**Conclusion:**

CT reports are a readily available and timely resource that may be exploited by NLP to facilitate continuous prospective IMD surveillance with translational benefits beyond surveillance alone.

## Introduction

Despite the high health and economic burden [1] of invasive mold diseases (IMDs) and effort invested in preventing them, prospective continuous epidemiological surveillance as advocated by professional societies and practice guidelines [2]–[4] is rarely performed. Prospective epidemiological surveillance in routine practice [5] is an onerous and costly task for hospitals principally because IMDs lack an easily identifiable and consistent laboratory prompt such as a positive blood culture. Case finding, like diagnosis, relies on a constellation of findings from clinical review in conjunction with radiology and microbiology with adjudication by experts using complicated case definitions [6] making it a time-consuming and therefore costly exercise that is not widely performed outside research protocols [7]–[10].

For surveillance in general, the primary screening method should have a high sensitivity in order to minimise the burden of case finding while maximising case capture. However, for epidemiological surveillance of IMDs the ideal screening method is undefined. Laboratory-based surveillance is subject to significant underreporting because IMDs may be diagnosed with or without microbiological confirmation corresponding to probable/proven and possible categories respectively [6]. Indeed possible infections may predominate in some settings [11]–[13] due to the difficulties in establishing a microbiological diagnosis given that conventional microbiology for *Aspergillus* and hyaline molds is positive in 50% or fewer of cases [14] and patient acuity often contraindicates invasive diagnostic procedures. Microbiological confirmation is further hampered by the fact that non-culture based tests (NCBTs) such as galactomannan (GM) or polymerase chain reaction (PCR) are not widely available and have a suboptimal sensitivity [15], [16] which is further compromised by concomitant antifungal therapy administered for either treatment or prophylaxis [17]. Administrative data such as coding diagnoses is unreliable for IMD surveillance [18] and neither timely nor informative enough for outbreak detection. In practice, no single method will be adequate for epidemiological surveillance of IMDs and complete case capture will require, the pooling of data from multiple sources [7], [8], [18]. However, the combination of laboratory based data with clinical or bed-side surveillance to best characterize total disease burden is not feasible for many centers to perform in real-time as it is a labour and time intensive task.

Although the optimal screening method for epidemiological surveillance of IMDs is undefined, the high frequency of pulmonary involvement makes chest computed tomography (CT) imaging an attractive target for screening. Chest CT is routinely performed when IMD is suspected as it is a non-invasive test that is widely available with results reported within hours rather than days and pulmonary involvement is present in 90% to 100% of patients with IA [7], [8], [10]. Although lung sampling and other laboratory indicators could be used for epidemiological surveillance and may upgrade possible cases to probable/proven categories they are not performed with the same frequency of CT. The major shortcomings of CT, however, are its poor specificity for IMDs [19] and extracting meaning from free-text reports.

We hypothesized that epidemiological surveillance of IMDs that is cost-effective and sustainable could be facilitated by technology. Natural language processing (NLP) is a computational method of analyzing human language that has detected several medical conditions [20]–[24] from the wealth of unstructured data in hospitals, with an accuracy comparable to human interpretation [24]–[26]. We developed a classifier that uses NLP based on machine learning techniques, to flag suspicious CT reports performed during routine clinical practice as a means of facilitating prospective IMD epidemiological surveillance in hospitals.

## Methods

### Study Design and Setting

This was a retrospective case-control cohort study of patients from three tertiary adult university-affiliated hospitals (Alfred Health, AH; Peter MacCallum Cancer Institute, PM; Royal Melbourne Hospital, RMH). AH and RMH operate statewide haematopoietic stem cell transplant (HSCT) services which collectively perform approximately100 allogeneic transplants/year. De-identified patient records were used and the human research ethics committees at each site who granted permission for the study, waived patient consent.

### Inclusion and Exclusion Criteria

Case and uninfected control patients from 2003 to 2011 inclusive were identified from previously completed clinical mycology studies [1], [12], [27], [28] (Ananda-Rajah et. al. Unpublished work: Prophylactic Effectiveness & Safety Of Intermittent Liposomal Amphotericin In High Risk Haematological Patients Abstract no M-280 51st Interscience Conference on Antimicrobial Agents and Chemotherapy ASM, Chicago 2011) pharmacy dispensing records, antimicrobial stewardship, HSCT, infectious diseases databases and microbiology records. Patients lacking CT scan reports were excluded, as were exclusively brain scans in order to focus detection of sino-pulmonary disease. Because detection of IMDs was the intent, patients with isolated candidaemia were excluded as laboratory-based surveillance is suitable for this infection.

### Clinical Data and Definitions

CT reports from haematology/HSCT patients with (i.e. cases) and without IMDs (i.e. controls) were manually downloaded from each hospital as de-identified text files. CT reports, from performance of the diagnostic scan and for 12 weeks thereafter (in order to evaluate radiological progression) performed during the clinical encounter were included. The clinical encounter was defined from admission to either discharge, death or transfer. Clinical information was extracted from aforementioned study datasets and hospital records. IMDs were classified according to consensus definitions by expert reviewers [6]. Date of diagnosis was defined as the first day of suspicious radiological abnormality for possible cases or for probable/proven cases, a positive microbiological or histopathological test.

### Development of the Reference Standard

A randomly selected convenience subset of reports from case and control patients were annotated at sentence and scan level by three infectious diseases physicians (MAR, KT, MS). The primary reviewer (MAR) annotated all case and control reports. Secondary review of case reports was undertaken by two physicians (KT, MS). The secondary reviewers served to validate the primary reviewer’s analysis through measures of agreement. Pre-specified annotation guidelines were refined with differences in opinion resolved by discussion at face-to-face meetings. An iterative process of annotation ensued with reports from case patients annotated twice or three times over, while those from control patients (from the outset regarded as less challenging to interpret) were annotated once by the primary reviewer (MAR).

For annotation, each sentence within a report and each report was treated as an independent observation, meaning that for sentence level annotation, sentences rather than specific words were coded according to the following contextual features: specificity for IMD (specific vs non-specific features e.g. macronodules, halo vs. infiltrates, ground-glass, consolidation); certainty (suggestive, equivocal or not supportive of IMD), directionality/change (negation, stable, resolution, progression); temporality (recent, past); alternative processes (e.g. pulmonary emboli, edema) and clinical alerts (i.e. urgent follow-up required). Scan level annotation referred to classification of the entire report as being either supportive or not for IMD with equivocal scans subsequently merged with supportive reports. Supportive reports had specific features of IMD including halo, nodule, cavity, focal mass, wedge-shaped lesions, bony sinus erosions. Negative reports had none of the above features while equivocal reports included infiltrate, consolidation, ground glass change, effusions or uncertainty by the reporting radiologist.

Importantly, the reference standard was informed by the opinion of clinician experts rather than radiologists given that the guiding principle was to flag reports of concern to end-users irrespective of the final diagnostic outcome.

### Development of the Classifier

Reports were classified in a binary fashion as being supportive or not supportive of IMD (i.e. positive/negative). A multi-class classification approach at sentence level was used: each sentence allocated one of a number of classes, with all sentence-level classifications subsequently informing the report level decision.

Text was analyzed as groups of words (“bag-of-words”), phrases (“bag-of-phrases”) and concepts (“bag-of-concepts”) [29] which were extracted from the annotated reports. The bag-of-words framework collates unordered sets of words, mapping dates and numbers to date and number features respectively. The bag-of-phrases framework uses phrases identified by MetaMap [29] corresponding to concepts from the Unified Medical Language System (UMLS) Metathesaurus. The version of MetaMap employed leverages the Negex tool [30] to determine negation of a concept (e.g., “… is not consistent with …”). The bag-of-concepts framework used Metathesaurus concepts mapped by MetaMap, noting that multiple phrases may map to the same concept.

We adopted a supervised machine learning approach experimenting with several machine learning algorithms including Support Vector Machines (SVM), Naïve Bayes, Random Forests, and Bayesian Nets, as implemented in the Weka 3.6.0 [31] and LibSVM 3.11 [32] toolkits. Briefly, supervised machine learning is a group of computational methods which use algorithms to automatically construct a model from labeled/annotated training data that is then used to predict classification in unseen/unlabeled examples [33]. Of the algorithms tested, SVM performed best and was selected for the final classifier.

Physician annotated reports were divided into development (n = 366) and held-out sets (n = 83), the latter annotated at scan level only. We used 10-fold cross validation on the development set with the optimal classifier identified from experiments, tested against the held-out set which served as an additional validation step. Cross-validation is an accepted method within the machine learning domain that maximizes limited gold standard data while minimizing the risk of overfitting associated with training on the test set [34].

### Statistical Analysis

The results of each manually annotated report was compared to the binary and probabilistic predictions of the classifier allowing calculation of sensitivity, specificity and receiver operating curve (ROC).

Hypothetical IMD prevalence rates of 5%, 10% and 20% were used to estimate positive and negative predictive values (PPV, NPV). Inter-annotator agreement was assessed using Cohen’s kappa coefficient, a chance corrected index of agreement [35]. All analyses used Stata 11.0 software (Stata Corp, College Station, Texas, USA).

## Results

### Patient Characteristics

A total of 147 patients were included in the annotated subset of 449 reports; 79 (54%) had IMDs and 68 (46%) were control patients (Table 1). Neutropenia (≤0.5×10^9^ cells/L) was present in 82% and 75% of clinical encounters among case and control patients respectively and was prolonged (median 18 and 19 days respectively). A history of HSCT was present in 46% and 52% of case and control patients, being allogeneic in 86% and 77% of patients respectively.

**Table 1:** Characteristics of patients with and without invasive mold diseases (IMDs).

Characteristic	IMD group n (%)	Control group n (%)
No. of patients	79	68
No. of clinical encounters^1^	79 (51)	75 (49)
Male gender	48 (61)	35 (51)
Age, mean (range) years	53 (20–89)	51 (18–89)
Underlying disease		
AML	32 (41)	35 (51)
ALL	14 (18)	14 (19)
Lymphoma	15 (19)	12 (16)
Chronic leukaemia	7 (8.9)	1 (1.3)
MDS/transformed MDS	6 (7.6)	2 (2.7)
Multiple myeloma	3 (3.8)	3 (4)
Other	2 (2.5)	5 (6.7)
Neutropenia (≤0.5 cells/L) present	65 (82)	56 (75)
Median duration of neutropenia (IQR), days	18 (8–45)	19 (5–39)
HSCT	36 (46)	39 (52)
Allogeneic	31/36 (86)	30/39 (77)
Autologous	5/36 (14)	9/39 (23)
Characteristics of IMDs, n = 79		NA
Probable/proven IMDs	33 (42)	
Possible IMDs	46 (58)	
Site of infection		
Lung	67 (85)	
Sino-pulmonary	3 (3.8)	
Sinus	2 (2.5)	
Hepatosplenic	2 (2.5)	
Disseminated	4 (5.1)	
Organism		
*Aspergillus fumigatus*	13	
Non-fumigatus *Aspergillus* species (*A. niger, A. flavus*)	4	
Fungal hyphae resembling *Aspergillus* species	3	
*Scedosporium* species	4	
Any positive PCR	2	
*Rhizopus* species	4	
Other molds (*Acrophialophora fusispora, Paecilomyces lilacinus*)	2	
*Candida glabrata* (co-infection with *S. Prolificans* fungaemia)	1	

Abbreviations: AML, acute myeloid leukemia; ALL, acute lymphoblastic leukemia; MDS, myelodysplastic syndrome; IQR, inter-quartile range; HSCT, haematopoietic stem cell transplant.

1 Clinical encounter defined from admission to either discharge, death or transfer and for up to 12-weeks after where applicable.

IMDs were probable/proven in 33/79 (42%) and possible infections in 46/79 (58%). Sinus and/or pulmonary disease occurred in 91% of case-patients. IA comprised 20/33 (61%) of microbiologically confirmed cases with rare molds including *Scedosporium* and *Rhizopus* species identified in 10/33 (30%).

### Characteristics of the Dataset of CT Reports

Overall, 1880 reports were retrieved from 527 patients (51% with IMDs) (Table 2). A total of 7083 sentences were annotated at sentence level according to pre-defined contextual features. Mean report length per hospital was 314, 211 and 126 words reflecting inter-institutional variations in reporting styles. Hospital A supplied 50% of annotated reports. The annotated dataset predominantly comprised chest 375/449 (84%) and sinus scans (alone or in combination with other sites) 28/449 (8.5%). In the annotated subset, there were 10 reports from 5 outpatients, all of who were control patients not subsequently diagnosed with IMD.

**Table 2:** Characteristics of the physician expert annotated and unannotated reports.

Characteristic	Annotated reports n (%)	Unannotated reports n (%)
No. of reports	449	1431
Held-out reports^1^	83 (18)	NA
No. of patients total	147	380
No. of IMD patients	79	191
No. reports from IMD patients	294 (65)	905 (63)
No. reports from control patients	155 (35)	526
Chest (alone or in combination with sinus, abdo/pelvis, brain etc)	375 (84)	865 (60)
Sinus (alone or brain-sinus, orbits, abdo/pelvis)	38 (8.5)	44
Other (abdo, abdo pelvis, liver, aorta, neck)	36 (8.0)	408
No. of reports according to study site		
Hospital A	226 (50)	713
Hospital B	131 (29)	422
Hospital C	92 (20)	296
No. of words per report according to study site		
Hospital A	211	229
Hospital B	126	128
Hospital C	314	348

Abbreviation: IMD, invasive mold disease.

1 Held out reports were annotated at scan level only as being supportive, unequivocal or negative for IMD.

Inter-annotator agreement between primary reviewer and the two secondary reviewers combined, was fair at sentence level for distinguishing certainty from equivocal/negative labels (K = 0.64) but improved at report level for the same classification (0.83) given that K levels ≥0.75 represent excellent agreement [35].

### Performance of the Classifier

**Figure 1:**
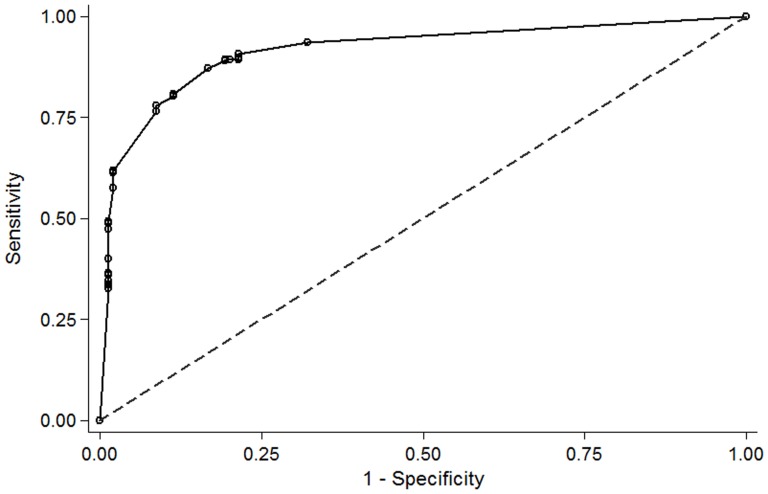
Receiver operating characteristic (ROC) curve for 321 inpatient reports comparing the probabilistic output of the classifier to expert opinion. Area under the ROC curve = 0.90 (95%CI 0.86 to 0.93). Abbreviation: CI, confidence interval.

Classifier performance among development and held-out sets respectively, was as follows (Table 3): sensitivity (i.e. concordance between classifier and physician annotation for reports supportive of IMD) was 91% (95%CI 86% to 94%)/88% (95%CI 74% to 95%); specificity was 79% (95%CI 71% to 84%)/70% (95%CI 55% to 81%); false negative rates were 20/366 (9.2%) and 5/83 (12.5%). The area under the ROC for the development set was 0.92 (95%CI 0.89 to 0.94) and 0.90 (95%CI 0.86 to 0.93) for the inpatient subset (n = 321) only (Figure 1). For the inpatient subset, sensitivity was 85% (95% CI 79% to 90%) and specificity was 86% (95% CI 81% to 93%). Using a sensitivity of 90% and specificity of 77% from the entire dataset of 449 reports at IMD prevalence’s of 5%, 10%, 20%, estimated PPVs were 17%, 30%, 49% and NPVs were 99%, 99%, 97%.

**Table 3:** Performance characteristics of the classifier.

Characteristic	TP	FP	TN	FN	Sn, % (95%CI)	Sp, % (95%CI)
Development dataset, reports n = 366	197	32	117	20	91 (86 to 94)	79 (71 to 84)
1Held-out dataset, reports n = 83	35	13	30	5	88 (74 to 95)	70 (55 to 81)
All reports, n = 449	232	45	147	25	90 (86 to 93)	77 (70 to 82)

1 Held out dataset were annotated at report level only as being positive, negative or equivocal for IMD.

Abbreviations: TN, true positives; FP, false positives; TN, true negatives; FN, false negatives; Sn, sensitivity; Sp, specificity; CI, confidence interval.

### Error Analysis

**Figure 2:**
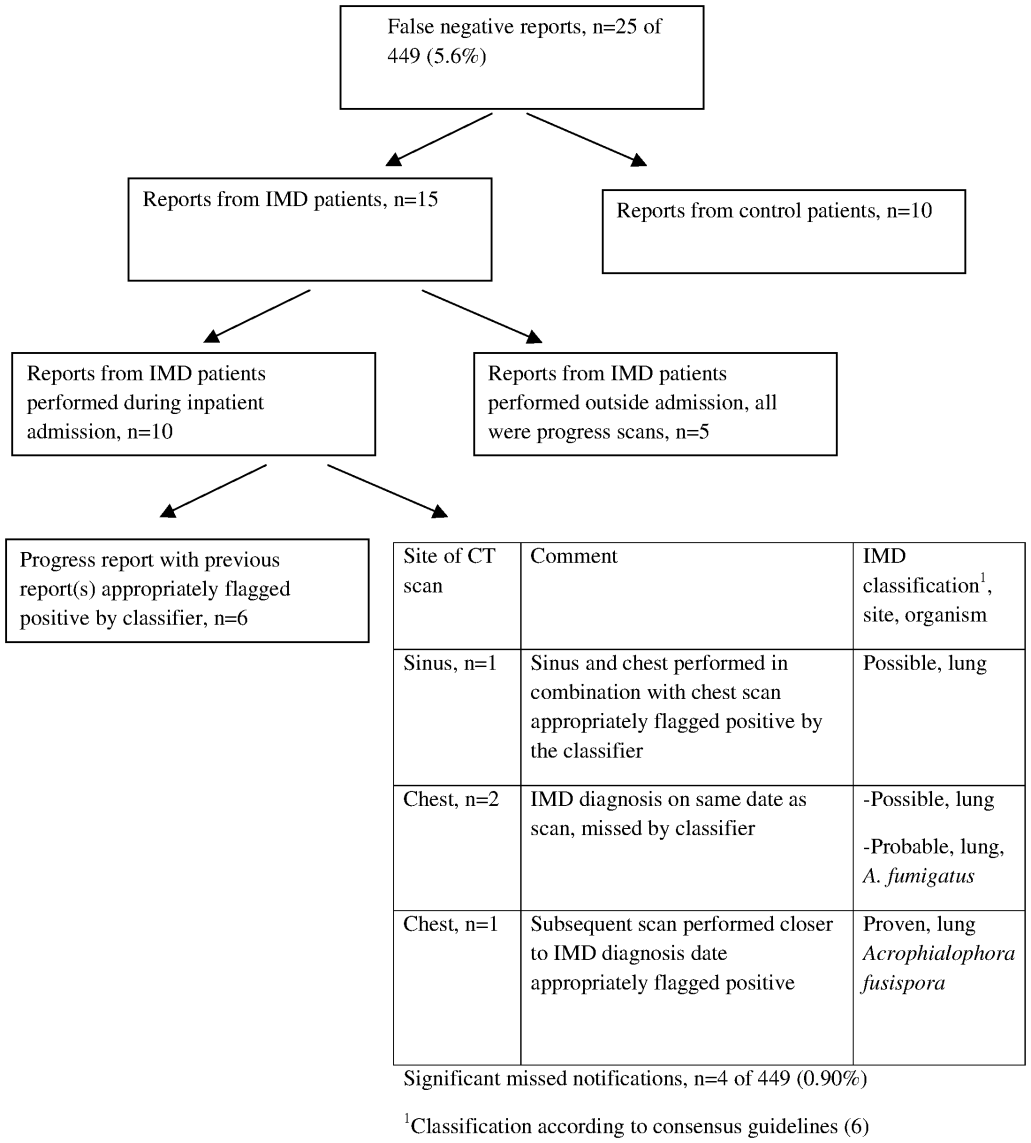
Error analysis of reports annotated supportive for invasive mold disease (IMD) but missed by the classifier. Abbreviations: CT, computed tomography.

Of the 25 missed cases (false negatives), 4 (4/449, 0.9%) were significant as shown in Figure 2. Reports from patients subsequently not diagnosed with IMD were disregarded (n = 10). The remaining 15 reports from case-patients comprised 10 inpatient reports including 6 progress reports whose antecedents were appropriately flagged. Five scans from case patients performed outside admission were follow up scans that provided information on clinical progress. Among the 4 significant missed notifications, one was a sinus scan in combination with a chest scan, the latter being flagged appropriately.

Review of 45 false positive reports revealed several sources of systematic error described in Table 4. Unsystematic errors were the result of three reports inappropriately annotated negative, two with sinus mucosal thickening and one describing ground glass pulmonary changes with a fungal aetiology entertained by the radiologist.

**Table 4:** Major systematic errors in the false notifications (false positives) for invasive mold diseases among computed tomography reports by the classifier.

Reason for misinterpretation	No. of reports	Characteristics
Inconsequential nodules	10	<1 cm nodules, granulomas
Abdominal scans	9	Non-specific hepatic or splenic lesions
Progress scans	9	Change in lesions rather than diagnosis the focus, therefore reports annotated negative by experts
Non-specific pulmonary/thoracic lesion	8	Atelectasis, scarring, mediastinal neoplastic mass
Misclassification	3	Pulmonary oedema, septic emboli, pulmonary lesions consistent with graft versus host disease

## Discussion

Mold infections are not well suited to prospective detection using manual methods of epidemiological surveillance due to the absence of an easily identifiable electronic prompt but may be rendered amenable to real-time detection using NLP of CT reports. Our classifier flagged reports suggestive of IMD from a variety of anatomic sites but overwhelmingly the sino-pulmonary tract (92%), the site most commonly involved by IA [8], [10], [36], achieving a sensitivity in the development subset, of 91% (95%CI 86% to 94%), specificity of 79% (95%CI 71% to 84%) and good overall accuracy with an area under the ROC of 0.92 [37]. Performance of the classifier was validated two ways, by cross-validation of development data in addition to held-out data, importantly with both methods using unseen data and producing similar findings (Table 3), lending robustness to the results.

For screening purposes, sensitivity is favoured over specificity especially for uncommon events like IMDs because missed cases are less tolerable than the resources spent following up false notifications [23]. The 25/449 (5.6%) missed notifications occurred at the expense of a modest number of false positive reports (45/449, 10%). Inpatient reports took precedence over the few outpatient reports given the higher clinical acuity of inpatients and the remote but real risk of nosocomial acquisition, resulting in a few missed notifications from case patients regarded as clinically significant (0.9%). False notifications were expected as the classifier was tuned for sensitivity, with inclusion of reports annotated equivocal included in the positive training bucket. Accordingly the classifier, like the physician annotators, was not designed to be conservative, assigning a positive label if there was any possibility of IMD.

In the absence of a gold standard for IMD reporting we relied upon peer review. Sentence level agreement between the primary and secondary annotator pair was fair (K = 0.64) despite measures mitigating unreliability including pre-specified guidelines, multiple experts and repeated consultation to resolve differences [35]. Our inter-annotator agreement is consistent with other cognitively challenging tasks such as ascribing pneumonia [38]–[40] or central-line associated blood stream infection [21] because deciding if clinical narratives are compatible with these complex conditions is sometimes difficult. Importantly, at report level, agreement was excellent (K = 0.83), noting that this endpoint (i.e. report rather than sentence level classification) is most relevant for the purpose of real-time surveillance or clinical decision support.

Our classifier has several limitations. Its poor PPV was not unexpected as PPV is highly conditional upon disease prevalence and, for uncommon events like IMDs, will be low despite a high sensitivity, as we observed [41]. High NPVs meant that a negative result could exclude IMD with some confidence. False notifications could potentially undermine confidence in a surveillance system and may be minimised (i.e. improving specificity) by including adjunctive sources of data (e.g. antifungal drug dispensing, microbiology) or by raising pre-test probability by filtering reports [41] based on clinical context (a clinical query of fever for example). It is possible but unlikely that changes in clinical practice over the long observational period of the study may have affected the radiological manifestations of IMD. The opinion of expert physicians rather than radiologists informed development of the classifier. However, these are the end users whose clinical acumen we sought to emulate and for similar syndromes such as pneumonia, albeit in chest radiograph reports, clinicians have demonstrated comparable performance to radiologists [25]. Annotation was unblinded, but informed by annotation guidelines that were developed in an iterative process. No conclusions can be drawn regarding classifier performance for subgroups with small numbers of reports such as sinus disease or hepatosplenic candidiasis. We confined ourselves to haematology-oncology population as this group is at highest risk for IMD [8], [42] and thus our findings may not be generalizable to other risk groups.

Further improvements in specificity may be achieved by omitting progress reports given their overrepresentation among false notifications. Experts often annotated progress reports negative because diagnostic radiological features may not always be re-iterated in a progress report. Non-specific pulmonary lesions such as atelectasis or scarring could be disregarded with the creation of handcrafted rules. Pulmonary nodules of questionable significance are more challenging to address, as size of lesions was not taken into account by the classifier. The development dataset did not exclusively comprise proven/probable-IMDs (despite these representing a higher degree of certainty) for several reasons: possible IMDs constitute a substantial burden in clinical practice and may predominate (up to 90%) in some centres [11], [12]; possible IMDs consume similar health-care resources (e.g. diagnostics, antifungal drugs) and their exclusion would underestimate true prevalence. Notably, all cases in our reference standard including possible-IMDs underwent expert adjudication according to international definitions [6]. Although we used a sample of reports from the entire dataset, the narrow confidence intervals around overall performance measures suggests that additional reports would not have made an appreciable difference. Finally, a dataset enriched with positive cases was used for classifier development yielding results acceptable for subsequent human verification but prospective validation of the classifier in the field is required.

The classifier is not a diagnostic adjunct but rather a screening tool designed to facilitate IMD case finding by exploiting routinely available clinical data [5], [7], [10]. The classifier’s strengths include its multi-site derivation; machine learning algorithms which unlike rule or knowledge based systems do not require manual programming of specific language features [43], [44] and consistency, by avoiding subjective interpretations of complicated case definitions [6].

Epidemiological surveillance for IMDs is needed for many reasons, including antifungal stewardship [45], clinical trial design, clinical audit, clinical registry development, intra and inter-facility comparisons. However, it is rarely performed in routine clinical practice, partly due to a lack of validated tools. Traditionally most IMD surveillance has relied on microbiological/histological diagnoses (which are not sensitive) with or without the addition of clinical diagnoses (which lack specificity). The intention of the classifier is to improve sensitivity i.e. case finding, with the advantage of early detection. This classifier is an additional tool to be used in combination with other methods to enable comprehensive surveillance of IMDs to be performed with minimal additional effort.
